# Use of initial and subsequent antihypertensive combination treatment in the last decade: analysis of a large Italian database

**DOI:** 10.1097/HJH.0000000000003215

**Published:** 2022-07-25

**Authors:** Laura Savaré, Federico Rea, Giovanni Corrao, Giuseppe Mancia

**Affiliations:** a National Centre for Healthcare Research & Pharmacoepidemiology, at the University of Milano-Bicocca, Milan, Italy; b MOX - Laboratory for Modeling and Scientific Computing, Department of Mathematics, Politecnico di Milano, Milan; c CHDS - Center for Health Data Science, Human Technopole, Milan; d Laboratory of Healthcare Research & Pharmacoepidemiology, Unit of Biostatistics, Epidemiology and Public Health, Department of Statistics and Quantitative Methods, University of Milano-Bicocca; e University of Milano-Bicocca, Milan, Italy

**Keywords:** antihypertensive treatment, drug combinations, healthcare utilization databases, population-based cohort studies

## Abstract

**Objective:**

The aim of the study was to assess the use of antihypertensive combination treatment, both as the initial and as a subsequent therapeutic step, in a large Italian population.

**Methods:**

The residents of the Lombardy Region (Italy), aged 40 years or older, who were newly treated with antihypertensive drugs during 2012, 2015 and 2018 were identified and the drug treatment strategy (monotherapy and combination of two, three and more than three antihypertensive drugs) was assessed at treatment initiation, and after 6 months, 1, 2, and 3 years of treatment. Data were also analysed after stratification for demographic and clinical categories.

**Results:**

About 100 000 patients were identified for each cohort. Monotherapy was the most common initial treatment strategy (75%), followed by two-drug single-pill combination (16%), two-drug free combination (6%), and combination of at least three drugs (3%). Use of two and three or more antihypertensive drugs increased during follow-up and reached about 32% (two drugs) and 11% (>2 drugs) of the patients after 3 years from treatment initiation. Among patients prescribed a two-drug combination, the single-pill was the most common approach, whereas the use of the three-drug single-pill combination was extremely rare. There were no substantial differences in the use of combination treatment between the three cohorts and the results were similar in all demographic and clinical categories.

**Conclusion:**

Our data show that in a real-life population use of antihypertensive drug combinations is low. They also show that, contrary to guideline recommendations, antihypertensive combination treatment did not show any noticeable increase in recent years.

## INTRODUCTION

Hypertension guidelines issued in the last 20 years have consistently recommended use of a combination of two or more antihypertensive drugs to treat most hypertensive patients [1–7]. This recommendation is strongly evidence-based. First, as hypertension is a multifactorial condition and blood pressure (BP) is a multiregulated variable [8], use of two or more antihypertensive drugs with complementary mechanisms of action is accompanied by greater BP-lowering effects and a much more frequent BP control than monotherapy [9,10]. Second, as BP reduction is associated with reduction of cardiovascular outcomes [11,12], a greater BP reduction reflects a greater cardiovascular protection, a conclusion recently documented by a meta-analysis of randomized outcome trials [13]. Third, as reported recently, compared with initial monotherapy, initial treatment with two antihypertensive drugs is associated with better adherence to the prescribed therapeutic regimen [14–16], lower therapeutic inertia [17], and as a result, more frequent long-term BP control and greater reduction of cardiovascular outcomes [18–20]. This has led latest guidelines to recommend use of two antihypertensive drugs not just, as in the past, after an ineffective monotherapy but as the initial treatment step [2,6,7].

Despite guideline recommendations, monotherapy has been reported to be still more frequently used than drug combinations in many countries [21–23], which implies that, rather the following the step-care approach recommended by guidelines (progressive addition of drugs to the initial one), doctors prefer to deal with an ineffective monotherapy by switching to another monotherapy, the so-called sequential monotherapy approach [21]. Aim of the present study was to determine the use of single, two, three or more antihypertensive agents in the population of Lombardy, a region of northern Italy with more than 10 million people. We analysed use of drug combinations both as the initial treatment step and over a 3-year treatment period. Data collection was extended to three temporal cohorts (2012, 2015 and 2018) to also determine whether mono versus combination treatment strategies had increased over the years. Because of the large number of patients involved, the analysis was also extended to different demographic and clinical categories.

## METHODS

### Setting

The data of the present study were retrieved from the Healthcare Utilization Databases of Lombardy, a region of Italy that accounts for about 16% (more than 10 million) of the entire Italian population. In Italy, all citizens have equal access to essential healthcare services provided by the National Health Service (NHS). In Lombardy, management of healthcare services is assisted by an automated system of databases that provides information on administrative data, outpatient drug prescriptions (according to the Anatomical Therapeutic Chemical or ATC system) and diagnosis at discharge from public or private hospitals (according to the *International Classification of Diseases*, 9th Revision, Clinical Modification or ICD-9-CM system). As in all the above-mentioned databases, patients are recorded via a single identification code, these databases can be interconnected, thereby providing the detailed healthcare pathway supplied to NHS beneficiaries. In order to preserve privacy, each identification code is automatically anonymized, the inverse process being only allowed to the Regional Authority upon request of judicial Authorities. Further details on Healthcare Utilization Databases in pharmacoepidemiological studies are available in previous studies [15–17,19,20].

### Cohort selection

The target population included the residents of Lombardy of both sexes who were aged 40 years or more and were beneficiaries of the NHS. Of these, residents who received at least one antihypertensive drug prescription during 2012 were identified, and the first drug dispensation was defined as the index prescription. An identical approach was used to recruit patients who received at least one antihypertensive drug prescription during 2015 and 2018. In each cohort, patients were included in the analysis if there was no prescription of antihypertensive agents in the previous 3 years, to limit the analysis to newly treated hypertensive individuals, the first prescription was followed by at least one other prescription, to avoid inclusion of patients with occasional prescriptions only, and at least 1 year of follow-up was available, to ensure enough time to complete the up-titration phase (which usually covers few weeks or months), and thus reach a stable treatment. The remaining patients were included in the final cohorts. The 2012 and 2015 cohort members were followed from the date of the index prescription until the earliest among the dates of emigration, death, or 3 years after the index prescription. The follow-up of 2018 cohort members was censored at 2 years after the index prescription because of the subsequent interference of the coronavirus disease 2019 (COVID-19) pandemic with outpatient prescription at the population level.

### Drug exposure

The complete list of drugs available for the treatment of hypertension in Italy is reported in Supplementary Table S1. All these antihypertensive drugs are provided free or almost free of charge by the Italian NHS upon prescription of a NHS doctor, which allows the databases to account for almost all antihypertensive drug use in the region. Among the patients who did not interrupt the drug treatment at each investigated time point, cohort members were classified according to the prescribed antihypertensive treatment strategy, that is, monotherapy, combination of two antihypertensive drugs in a single tablet or separately, combination of three antihypertensive drugs in a single tablet or separately, and combination of more than three antihypertensive drugs. In the 2012 and 2015 cohorts, antihypertensive treatment strategies were assessed at the date of index prescription (the initial strategy) and after 6 months, 1, 2 and 3 years. Follow-up was limited to 2 years for the 2018 cohort to avoid data overlapping with the year of Covid-19 pandemic and reduced prescription for all other diseases.

### Covariates

Baseline characteristics included sex, age, signs of cardiovascular disease (i.e. previous hospitalization for cardiovascular disease or prescription of one of the following drugs: antiarrhythmics, antiplatelets, anticoagulants, digitalis, and nitrates), co-treatments for metabolic cardiovascular risk factors (lipid-lowering and antidiabetic agents), antidepressants and drugs for pulmonary diseases. In addition, the number of co-medications dispensed during the 2 years before the index prescription date was assessed and categorized as 0, 1, 2, 3, 4, and at least 5. Finally, the clinical status of the patients was quantified by the Multisource Comorbidity Score, a prognostic score that has been shown to predict all-cause mortality and hospitalization of the Italian population better than some widely used conventional scores (i.e. Charlson, Elixhauser, and Chronic Disease scores) [24]. Four categories of clinical status were considered: good (score = 0), medium (1≤score≤4), poor (5≤score≤14) and very poor (score ≥15). Data were also separately analysed for residents in cities (the capitals of the 12 Lombardy provinces) and residents outside cities, that is, in less densely populated areas.

### Data analysis

Summary statistics of the prescribed antihypertensive treatment strategy, both at the date of index prescription and during follow-up, were expressed as counts and percentages. Standardized mean differences were used whenever appropriate to test differences between cohorts of the calendar years. Equipoise was considered to be reached when the between-group comparison of variables had a mean standardized difference of less than 0.1 [25].

The Statistical Analysis System Software (version 9.4; SAS Institute, Cary, North Carolina, USA) was used for the analyses.

## RESULTS

### Patients

About 100 000 patients fulfilled the inclusion criteria for each cohort. The clinical characteristics of the patients according to the calendar year are shown in Table 1. About 4 out of 10 patients were aged at least 65 years, half of them were women, one in four patients had cardiovascular disease, one in seven patients was co-treated with lipid-lowering drugs, and 1 in 14 patients with antidiabetic agents. One in four patients was prescribed at least five co-medications, and one in six patients had a poor clinical status. Except for the prevalence of cardiovascular disease (somewhat greater in the 2012 cohort), there were no substantial differences in demographic and clinical variables between the three cohorts.

**TABLE 1:** Baseline characteristics of cohort members according to the year of recruitment

	Year of recruitment			
	2012 (*N* = 100 252)	2015 (*N* = 100 050)	2018 (*N* = 103 225)	SMD
Age (years)				
40–64	60 238 (60.1%)	59 650 (59.6%)	62 366 (60.4%)	0.000
65–79	31 951 (31.9%)	31 976 (32.0%)	31 992 (31.0%)	0.000
80–89	7222 (7.2%)	7419 (7.4%)	7845 (7.6%)	0.024
≥90	841 (0.8%)	1005 (1.0%)	1022 (1.0%)	0.087
Sex				
Male	51 559 (51.4%)	51 225 (51.2%)	52 682 (51.0%)	0.000
Female	48 693 (48.6%)	48 825 (48.8%)	50 543 (49.0%)	0.000
CV disease^a^				
Yes	25 667 (25.6%)	23 173 (23.2%)	21 122 (20.5%)	0.123
No	74 585 (74.4%)	76 877 (76.8%)	82 103 (79.5%)	0.123
Co-treatments				
Lipid-lowering	14 156 (14.1%)	14 783 (14.8%)	16 259 (15.8%)	0.049
Anticoagulant/antiplatelet	14 288 (14.3%)	11 891 (11.9%)	9759 (9.5%)	0.197
Antidiabetic	7230 (7.2%)	6718 (6.7%)	6761 (6.6%)	0.042
Antidepressant	14 249 (14.2%)	14 014 (14.0%)	14 099 (13.7%)	0.024
Drugs for lung diseases	24 143 (24.1%)	25 270 (25.3%)	26 630 (25.8%)	0.024
Number of co-medications^b^				
0	14 146 (14.1%)	14 327 (14.3%)	14 842 (14.4%)	0.000
1	16 908 (16.9%)	16 611 (16.6%)	17 498 (17.0%)	0.000
2	17 036 (17.0%)	16 442 (16.4%)	17 421 (16.9%)	0.024
3	15 427 (15.4%)	15 028 (15.0%)	15 228 (14.8%)	0.024
4	12 212 (12.2%)	12 027 (12.0%)	12 236 (11.9%)	0.000
≥5	24 523 (24.5%)	25 615 (25.6%)	26 000 (25.2%)	0.025
Clinical status^c^				
Good	42740 (42.6%)	41 877 (41.9%)	44 012 (42.6%)	0.000
Medium	40634 (40.5%)	41 430 (41.4%)	42 926 (41.6%)	0.000
Poor	13607 (13.6%)	13 651 (13.6%)	13 391 (13.0%)	0.024
Very poor	3271 (3.3%)	3992 (4.0%)	2896 (2.8%)	0.135

CV, cardiovascular; SMD, standardized mean difference.

a Hospital admission for cardiovascular disease or use of selected drugs (antiarrhytmics, antiplatelets, anticoagulants, digitalis or nitrates)

b Number of drugs with different three-digit ATC dispensed in the previous 2 years.

c Clinical status was assessed by the MCS, and four categories were considered: good (score = 0), medium (score ≥1 to ≤4), poor (score ≥5 to ≤14) and very poor (score ≥15).

### Initial drug treatment strategies

**FIGURE 1:**
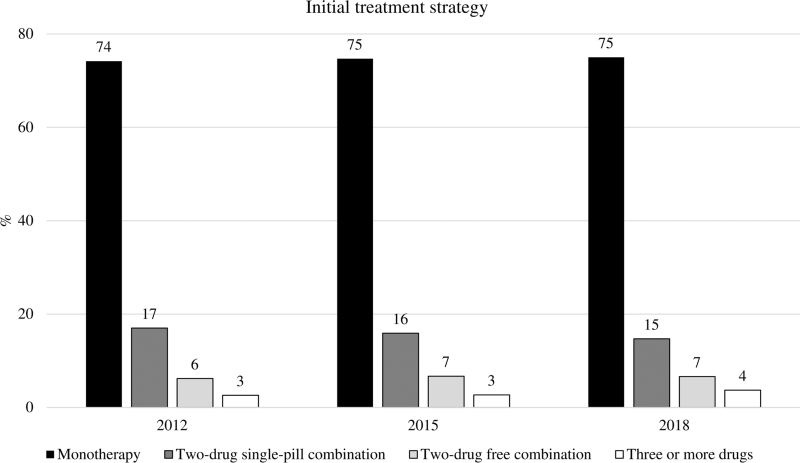
Initial drug treatment strategy, that is, monotherapy, two-drug combination (in a single tablet or separately), and three or more drug combination.

Figure 1 shows the prevalence of the different treatment strategies at the index prescription, that is, at treatment initiation. Monotherapy was by far the most common initial treatment strategy (approximately three out of four patients), with no significant differences between the 2012, 2015 and 2018 cohorts (standardized difference <0.1). Initial two-drug combinations involved only about one out of five patients, again with no between-cohort differences. Initial prescription of two-drug single-pill combinations markedly and similarly exceeded that of free combinations in all cohorts. Initial prescription of three or more drugs occurred in 3–4% of the patients

**FIGURE 2:**
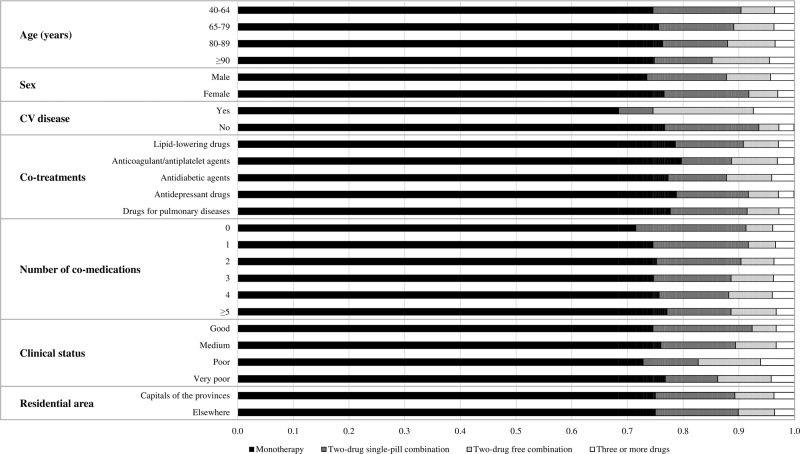
Percentages of patients starting antihypertensive drug treatment with one drug, two drugs (either single-pill or free combination) and three or more drugs in 2018 according to baseline characteristics.

The results were substantially similar in all strata of age, sex, clinical status, co-treatments, number of co-medications and evidence of cardiovascular disease (Fig. 2). No differences were observed also comparing the residents in the capitals of the provinces of the region versus those living in areas with less population density. Use of initial combination therapy was lowest among patients treated with anticoagulant/antiplatelet agents or drugs for metabolic cardiovascular risk factors (about 20%) whereas it was the highest among patients with cardiovascular disease (>30%). Use of initial three or more drug combinations was more common in older patients and in patients with a previous cardiovascular event compared with those with no cardiovascular events (about 6 versus 2%).

### Drug treatment strategies during follow-up

**FIGURE 3:**
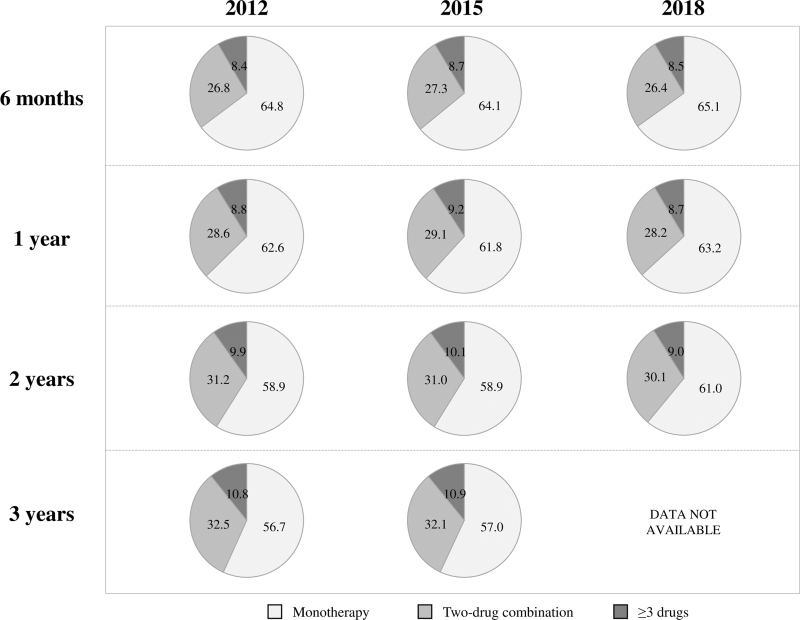
Percentages of patients under different drug treatment strategies at the sixth month and at the first, second, and third year after the initial prescription date.

Figure 3 shows that use of two-drug combinations increased progressively from 6 months after treatment initiation onward, to reach about 32% of the patients after 3 years, similarly in the 2012 and the 2015 cohorts. Use combinations of three or more drugs also increased progressively in both the 2012 and the 2015 cohorts, reaching in both cohorts about 11% after 3 years. Thus, after 3 years, combination treatment involved approximately 43% of either cohort. Similar findings were obtained for the 2018 cohort up to the 2 years of follow-up. The results were substantially similar in all demographic and clinical strata, again with the highest use of drug combinations in patients with cardiovascular disease (Table 2).

**TABLE 2:** Drug treatment strategy among patients with cardiovascular disease at the sixth month and at the first, second, and third year after the initial prescription date

	**Six months**		
	**2012**	**2015**	**2018**
Monotherapy	60.6%	59.1%	60.5%
Two-drug combination	28.6%	29.0%	27.4%
At least three drugs	10.8%	11.9%	12.1%
	**One year**		
	**2012**	**2015**	**2018**
Monotherapy	59.4%	57.5%	59.7%
Two-drug combination	29.4%	29.9%	28.9%
At least three drugs	11.2%	12.6%	11.4%
	**Two years**		
	**2012**	**2015**	**2018**
Monotherapy	56.4%	55.7%	58.7%
Two-drug combination	31.5%	31.4%	29.6%
At least three drugs	12.1%	12.9%	11.7%
	**Three years**		
	**2012**	**2015**	
Monotherapy	55.0%	54.8%	
Two-drug combination	32.2%	31.9%	
At least three drugs	12.8%	13.3%	

**FIGURE 4:**
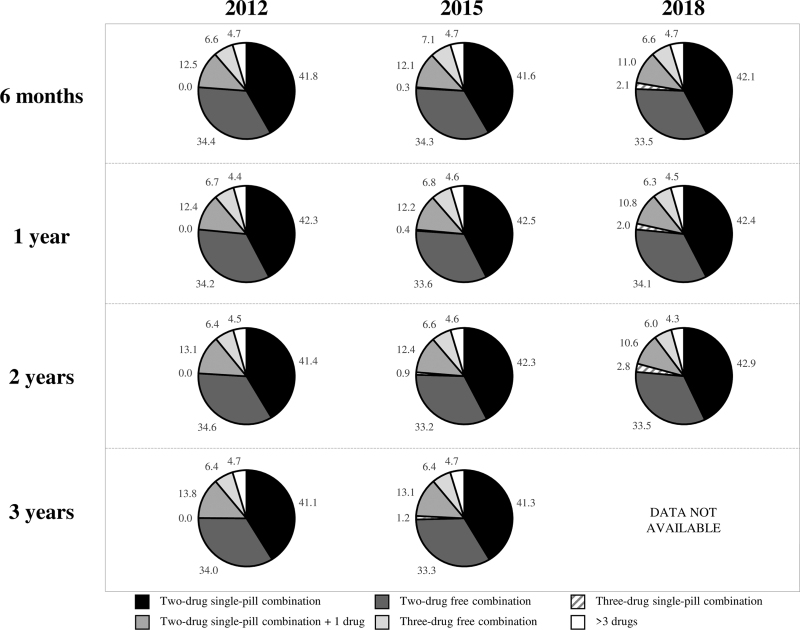
Use of free versus single-pill combinations among patients prescribed a combination therapy at the sixth month and at the first, second, and third year after the initial prescription date.

The drug treatment strategies used by patients prescribed a combination therapy during the follow-up are shown in detail in Fig. 4. About 75% of patients under combination treatment made use of a two-drug combination, with a large prevalence of single-pill compared with free drug combinations. Three drugs were used in about 20% of the patients under combination treatment, with a clear prevalence of those using two drugs in a single pill and one separately. Use of single-pill three-drug combinations was extremely rare over the entire follow-up whereas more than three drugs were used in less than 5% of the patients. There were no substantial differences in the cumulative use of combination treatment between the three cohorts, except for an appreciable increase in the use of single-pill three-drug combinations at the second year in the 2018 cohort.

## DISCUSSION

Our study shows that, in the Lombardy population, initial treatment with a two antihypertensive drug combination involved approximately one out of four patients, thereby being largely minority compared with initial monotherapy. It further shows that drug combinations were used less frequently than monotherapy also during chronic antihypertensive treatment as although progressively increasing from initial monotherapy, after 3 years of treatment, use of two or more antihypertensive drugs involved only about 43% of the patients. It finally shows that both initial and subsequent combination treatment did not exhibit any substantial increase from 2012 to 2018. This leads to the conclusion that, in this large fraction of the general Italian population, antihypertensive treatment shows a persistent departure from guidelines recommendations, which strongly support the use of drug combinations in most hypertensive patients based on the evidence that two or more antihypertensive drugs lower and control an elevated BP much more effectively than monotherapy [9,10]. As use of drug combinations during chronic antihypertensive treatment has been supported by guidelines over a long time, this also implies that recommendations of hypertension guidelines have a limited influence on Italian clinical practice.

Several other results of our study deserve to be mentioned. First, the limited use of antihypertensive drug combinations, initially or later during chronic treatment, involved all demographic and clinical patient categories, that is male and female individuals, patients of different ages, patients with different comorbidities, patients taking a different number of daily tablets, patients with different clinical conditions, and patients living in more (capitals of the Lombardy provinces) or less densely populated areas. Some differences are noteworthy, however. For example, there was a somewhat larger use of combination treatment, both initially and at the third year of treatment, in patients with a history of cardiovascular disease. Second, two-drug single-pill combinations were used more frequently than free two-drug combinations, this being the case in all demographic and clinical subgroups and for both initial and subsequent treatment. Third, during chronic treatment, three drugs were used in about 11% of the patients. Although guidelines remain somewhat vague about how many patients may require three-drug combinations to achieve BP control [2,6,7], clinical studies speak in favour of a substantially greater percentage [26], which is probably going to further increase after the recommendation of the most recent guidelines to try to achieve lower BP targets than in the past [2,6,7]. Fourth, in contrast with the larger use of two-drug single-pill versus free combinations, in each cohort initial and subsequent use of three-drug single-pill combinations was much lower than use of free three-drug combinations and in absolute numbers negligible. Thus, although single-pill combinations of three antihypertensive drugs are available in the Italian market since 2015, this treatment strategy does not appear to have made its way into clinical practice. It should be mentioned, however, that in the 2018 cohort, use of the single-pill three-drug combinations did show an increase from the previous levels after 2 years of treatment. Finally, a small but clearcut percentage of patients made use of a free three-drug combination initially, which is against the recommendations of the guidelines to never start treatment with three antihypertensive agents to avoid the risk of excessive BP reductions and injurious falls. In this context, an apparently paradoxical finding was that initial three-drug combination treatment was more common in very elderly patients (≥80 or 90 years of age) than in younger patients. This may at least in part result from the greater prevalence, in the very elderly stratum of the population, of conditions, such as heart failure or coronary disease in which antihypertensive drugs are required for their BP-independent direct organ protective properties and given to the patients initially rather than added progressively during a titration phase.

Evidence is available that low use of combination treatment is majorly involved in the low rate of BP control that characterizes the hypertensive population worldwide [27] as drug combinations are necessary to effectively reduce an elevated BP in most hypertensive patients [1–3,19,21]. How to make drug combinations the most common antihypertensive treatment strategy is beyond the aim of the present study. However, factors that might substantially improve on the present situation are a capillary educational campaign to increase patients’ awareness of the risk of hypertension on one side as well as of the protective effect of treatment on the other; a greater familiarity of physicians with international or national hypertension guidelines; a more frequent relationship between doctor and patient to be obtained also by implementation of telemedicine facilities [28] and a more favourable attitude of health authorities on drug combinations, including those recommended by guidelines as first step treatment and available as single pill formulation. For economic reasons, regulatory authorities sometimes are against these recommendations with little consideration for the evidence that both initial and single-pill combinations improve adherence to treatment [16], reduce inertia [17], favour BP control [29] and in observational studies show a reduction in cardiovascular events [18,23]. The guiding concept should be that a substantially better BP control via greater use of combination treatment is a priority goal in medicine as hypertension is the first cause of death worldwide [30].

The present study has several elements of strength. First, our investigation was based on a very large unselected population as our database involved all residents of the region. Second, as patients with antihypertensive drug prescriptions in the previous 3 years were excluded from analysis, those included were presumably newly treated individuals. This ‘new user’ approach reduced the potential for selection bias and confounding [31]. Third, the drug prescription database provided highly accurate data as pharmacists are required to report prescriptions in detail in order to obtain reimbursement, and incorrect reports have legal consequences.

Our study has also limitations. First, the antihypertensive drugs prescribed in the context of private visits are not included in the Lombardy database. However, in Italy, the availability of free medical care makes use of private medicine rare, that is, about 6% of the drugs used for cardiovascular diseases [32]. This is the case also for all the other drugs included in our analysis except for aspirin whose large over the counter availability grossly underestimated use of antiplatelet drugs. Second, as mentioned above as antihypertensive drugs are also prescribed for heart failure and coronary disease, our data may not exclusively reflect antihypertensive treatment, which is nevertheless by far the most common clinical condition for which the drugs we have analysed are prescribed [33]. Third, although use of combination treatment was analysed in patients with a variety of different demographic and clinical characteristics, our database did not allow to extend the analysis to socioeconomic factors, and thus to determine whether the economic status, educational levels, employment status play a role in the acceptance of guidelines recommendation to largely base antihypertensive treatment on drug combinations. Furthermore, factors, such as use of combination treatment in rural areas could not be precisely addressed as Lombardy is a highly urbanized region, which limited our analysis to residents in more versus less densely populated areas. Finally, our database does not include BP values, which prevents our investigation to relate use of mono and combination treatment strategies to the achievement of BP control.

In conclusion, our study shows that, despite the long-term recommendations of hypertension guidelines, in real life use of combination treatment is low and little improvement appears to occur with time. It further shows that this is the case for both initial and subsequent use of drug combinations. As drug combinations guarantee a much higher rate of effective BP reduction and control, this has detrimental consequences for the prevention of cardiovascular diseases.

## ACKNOWLEDGEMENTS

Funding: this study was supported by grants from the Italian Ministry of the Education, University and Research (’Fondo d’Ateneo per la Ricerca’ portion, year 2019), and from the Italian Ministry of Health (‘Ricerca Finalizzata 2016’, NET- 2016-02363853). The funding sources had no role in the design of the study, the collection, analysis and interpretation of the data, or the decision to approve publication of the finished manuscript.

### Conflicts of interest

Disclosures: G.C. received research support from the European Community, the Italian Medicines Agency, the Italian Ministry of Education, University, and Research, and the Italian Ministry of Health. He took part to a variety of projects that were funded by pharmaceutical companies (i.e. Novartis, GlaxoSmithKline, Roche, AMGEN, and Bristol-Myers Squibb). He also received honoraria as member of Advisory Board from Roche. G.M. received honoraria for participation as speaker/chairman in national/international meetings from Boehringer Ingelheim, Ferrer, Medtronic, Menarini Int, Merck Serono, Recordati, Servier and Sanofi. The other authors report no conflicts.

## Supplementary Material

Supplemental Digital Content
