# Supplementary material for: Use of initial and subsequent antihypertensive combination treatment in the last decade: analysis of a large Italian database
Source: J Hypertens. 2022 Jul 25;40(9):1768–75. doi: 10.1097/HJH.0000000000003215 (PMC10860882; doi:10.1097/HJH.0000000000003215)
Supplement: Supplemental Digital Content [file jhype-40-1768-s001.docx]

**Supplementary Table S1**. Antihypertensive drugs available in the Italian market

| **Antihypertensive drugs** |
| --- |
| **Monotherapy** |
| ACEIs |
| ARBs |
| β-blockers |
| Diuretics |
| CCBs |
| Alpha-blockers |
| Renin-inhibitors |
| **Two-drug single-pill combination** |
| ACEI + Diuretic |
| ACEI + CCBs |
| ARBs + Diuretic |
| ARBs + CCBs |
| β-blocker + Diuretic |
| ACEI + β-blocker |
| **Three-drug single-pill combination** |
| ACEI + CCBs + Diuretic |

ACEIs: angiotensin-converting enzyme inhibitors; ARBs: angiotensin receptor blockers; CCBs: calcium-channel blockers
